# Immune Checkpoints Expression in Chronic Lung Allograft Rejection

**DOI:** 10.3389/fimmu.2021.714132

**Published:** 2021-08-13

**Authors:** Ilaria Righi, Valentina Vaira, Letizia Corinna Morlacchi, Giorgio Alberto Croci, Valeria Rossetti, Francesco Blasi, Stefano Ferrero, Mario Nosotti, Lorenzo Rosso, Mario Clerici

**Affiliations:** ^1^Thoracic Surgery and Lung Transplantation Unit, Fondazione IRCCS Ca’ Granda-Ospedale Maggiore Policlinico, Milan, Italy; ^2^Division of Pathology, Fondazione IRCCS Ca’ Granda-Ospedale Maggiore Policlinico, Milan, Italy; ^3^Department of Pathophysiology and Transplantation, University of Milan, Milan, Italy; ^4^Respiratory Unit and Adult Cystic Fibrosis Center, Internal Medicine Department, Fondazione IRCCS Ca’ Granda-Ospedale Maggiore Policlinico, Milan, Italy; ^5^Department of Biomedical, Surgical and Dental Sciences, University of Milan, Milan, Italy; ^6^Don C. Gnocchi Foundation, IRCCS, Milan, Italy

**Keywords:** lung transplant, chronic rejection, immunology, Treg lymphocytes, PD-1 and PD-L1, FoxP3

## Abstract

Chronic lung allograft dysfunction (CLAD) is the main cause of poor survival and low quality of life of lung transplanted patients. Several studies have addressed the role of dendritic cells, macrophages, T cells, donor specific as well as anti-HLA antibodies, and interleukins in CLAD, but the expression and function of immune checkpoint molecules has not yet been analyzed, especially in the two CLAD subtypes: BOS (bronchiolitis obliterans syndrome) and RAS (restrictive allograft syndrome). To shed light on this topic, we conducted an observational study on eight consecutive grafts explanted from patients who received lung re-transplantation for CLAD. The expression of a panel of immune molecules (PD1/CD279, PDL1/CD274, CTLA4/CD152, CD4, CD8, hFoxp3, TIGIT, TOX, B-Cell-Specific Activator Protein) was analyzed by immunohistochemistry in these grafts and in six control lungs. Results showed that RAS compared to BOS grafts were characterized by 1) the inversion of the CD4/CD8 ratio; 2) a higher percentage of T lymphocytes expressing the PD-1, PD-L1, and CTLA4 checkpoint molecules; and 3) a significant reduction of exhausted PD-1-expressing T lymphocytes (PD-1^pos^/TOX^pos^) and of exhausted Treg (PD-1^pos^/FOXP3^pos^) T lymphocytes. Results herein, although being based on a limited number of cases, suggest a role for checkpoint molecules in the development of graft rejection and offer a possible immunological explanation for the worst prognosis of RAS. Our data, which will need to be validated in ampler cohorts of patients, raise the possibility that the evaluation of immune checkpoints during follow-up offers a prognostic advantage in monitoring the onset of rejection, and suggest that the use of compounds that modulate the function of checkpoint molecules could be evaluated in the management of chronic rejection in LTx patients.

## Introduction

Lung transplantation (LTx) is a valuable therapeutic choice for selected patients with end-stage respiratory failure. Unfortunately, LTx has a relatively poor long-term prognosis, considering that 6.7 years is the median survival of patients transplanted between 2010 and 2017 (1). The most common cause of this high mortality is chronic lung allograft dysfunction (CLAD), a clinical condition characterized by progressive and irreversible decline in lung function, which leads to retransplantation or, more often, death. CLAD includes at least two well described clinical entities: bronchiolitis obliterans syndrome (BOS) and restrictive allograft syndrome (RAS), the latter being associated with the worst prognosis. BOS and RAS have different functional pictures, pathological figures, and radiological findings (2); their pathogenesis is poorly understood and a specific therapy is not available yet. Although the mechanisms leading to BOS or RAS are unknown, similar pathways involving innate immunity, antibody-mediated rejection, and cellular rejection are likely to be responsible for their pathogenesis.

In solid organ transplantation, lungs are the only grafts that are open to external environment: air pollution, bacteria, and viruses can directly damage the recipient alveolar cells and bronchial epithelium, and activate dendritic cells. Therefore, activation of the innate and the adaptive immune responses makes the lung graft a peculiar local environment (3, 4). Several studies have addressed the role of dendritic cells, macrophages, T cells, donor specific antibody, anti-HLA antibodies, and interleukins in chronic rejection and CLAD (5–7), but the possible role of immune checkpoint molecules expression and function in this phenomenon has not yet been analyzed. Sporadic experiences with immune checkpoint inhibitor treatments of kidney and heart transplanted patients suffering from neoplastic diseases have shown that the use of these molecules results in the rapid development of severe rejection (8). These observations underline the need to better understand the possible role of immune checkpoint molecules in transplantation, and in particular in LTx. Takahashi and co-workers demonstrated in a LTx animal model the specific behavior of CD8^pos^ T lymphocytes in inducing tolerance. The absence of PD-1 on such lymphocytes was observed to favor the creation of prolonged interactions between CD8^pos^ T cells and CD11c^pos^ graft-infiltrating dendritic cells. This favored the differentiation of CD8^pos^ T lymphocytes into effector memory phenotype, resulting in acute graft rejection (9). We conducted the present retrospective pilot observational study on lungs explanted during re-transplantation (re-LTx) in patients with BOS or RAS to gain new insights into the possible role of immune checkpoint in lung allograft tolerance.

## Materials and Methods

We conducted an observational pilot retrospective study on lung grafts explanted during re-LTx for CLAD in our institution (Foundation IRCCS Ca’ Granda-Ospedale Maggiore Policlinico of Milan) in eight consecutive recipients between 2014 and 2019 (**Table 1**). As controls, we used six normal lung parenchyma from patients surgically resected for lung cancer.

**Table 1:** Clinical characteristics of lung re-transplant recipients^1^.

Patient	Sex	Age (years)	Disease at 1st LTx	Freedom from CLAD (months)	Time from 1st LTx (months)	CLAD grade	RAS (Yes/No)
Re-LTx #1	F	23	CF	13	38	3	Y
Re-LTx #2	M	38	CF	23	74	4	Y
Re-LTx #3	F	25	CF	14	24	4	Y
Re-LTx #4	M	36	LCH	14	19	4	Y
Re-LTx #5	F	34	CF	25	34	4	N
Re-LTx #6	F	31	CF	10	32	4	N
Re-LTx #7	F	27	CF	46	27	4	N
Re-LTx #8	F	32	CF	33	150	3	N

^1^BOS, Bronchiolitis Obliterans Syndrome; CF, Cystic Fibrosis; CLAD, Chronic Lung Allograft Disease; LCH, Pulmonary Langerhans Cell Granulomatosis - Histiocytosis X; RAS, Restrictive Allograft Syndrome.

The pathologists (SF, GC, and VV) were blinded to the clinical course of patients. The Hospital Institutional Review Board approved the study (ref. 1693/2018), and all patients signed a written consent for biobanking of blood and tissues samples. At the enrollment in the present study, all the clinical cases were carefully reviewed by checking functional parameters, medical history, and pathological reports: two different clinicians, blinded to each other and to the patients’ identity, confirmed the diagnosis of RAS in four patients and grade 3–4 BOS in the remaining four according to pulmonary function and ISHLT radiological criteria (10, 11). The patients’ clinical history included treatment with pulse steroid and/or steroid taper on whenever any grade of acute rejection was found on transbronchial biopsy at CLAD onset; subsequently, they all underwent antiproliferative switch (i.e., from azathioprine to mycophenolate), and all but one started extracorporeal photo-pheresis (ECP; the only exception was a patient who was too fatigued to be treated with ECP). Finally, these patients were considered for re-LTx when gas exchange deteriorated both on exertion and at rest; one patient was bridged with extracorporeal membrane oxygenation support and two patients were prioritized due to respiratory failure with high oxygen flow at rest and non-invasive ventilation dependency.

Representative 4-μm-thick sections were cut from each block from explanted lungs (two blocks per case) and stained as previously described (12). Positive and negative controls were included in each experiment. Single staining was revealed using DAB as chromogen, whereas for double immunohistochemistry the antibodies were colored as previously described (13). All slides were counterstained with hematoxylin and digitalized using Aperio scanner at 40x magnification (Leica Microsystems). Presence of staining for all antibodies was evaluated only in the lymphocytic infiltrates. Forkhead box P3 (Foxp3), programmed cell death protein 1 (PD1), and CD4-, CD8-, or Pax5-expressing cells were quantified using a nuclear (Foxp3) or a cytoplasmic specific algorithm (Genie Histology Pattern Recognition software; Leica Microsystems). Programmed death-ligand 1 (PDL1), cytotoxic T-lymphocyte antigen 4 (CTLA4), and T-cell immunoglobulin and ITIM domain (TIGIT) scoring was performed manually. Positivity for thymocyte selection-associated, high-mobility group box (TOX) was only scored in conjunction with CD4, CD8, Pax5, or PD1 in double immunohistochemical (IHC) staining. Detail of the antibodies is provided in **Supplementary Table 1**.

Briefly, three pathologists (VV, GC, and SF) independently analyzed the slides from all patients and selected the two most representative per case. Then, four regions per slide were analyzed for markers presence. The mean number of counted cells per each marker is indicated in **Supplementary Table 2**.

The digital algorithms used to score the immunophenotypic markers (CD3, CD4, CD8, and Pax5) as well as FOXP3 and PD1 were previously validated (12). For the remaining antibodies for which a digital scoring was not possible because of high background/weak signal, the three pathologists (VV, GC, and SF) independently reviewed the slides and agreed on a dichotomous score (positive or negative) using as threshold at least 5% of positive lymphocytes.

For double IHC, the two signals were split using the Aperio ePathology Image Quality (IQ) color processing; cells stained with the first antibody were identified (circled) and analyzed for the presence of the second marker. Data are expressed as percentage of double-positive cells/percentage of cells that expressed the first protein. For each section, at least 1,000 lymphocytes were analyzed.

Clinical data were summarized as absolute and percentage or median and range or 95% confidence interval, as appropriate. IHC data are presented as percentages of positive cells and summarized using individual value plots with median and interquartile range (IQR), unless otherwise specified. Samples were compared using the two-sided non-parametric Mann-Whitney U test. For categorical analyses, the number of patients in each category is shown, and data were analyzed using Chi-square or Fisher exact test as appropriate. Analyses were performed using MedCalc (MedCalc Software Ltd, Ostend, Belgium) or R studio (version 3.2.2), and charts were generated with GraphPad Prism software (San Diego, CA, USA).

## Results

**Table 1** summarizes the clinical parameters of re-LTx recipients included in the current case series. Briefly, six of the patients were female, median age was 31.5 years (95% C.I. from 24.6 to 36.4 years), and the median free-from-CLAD time was 18.5 months (95% C.I. from 12.4 to 35.5 months).

**Figure 1:**
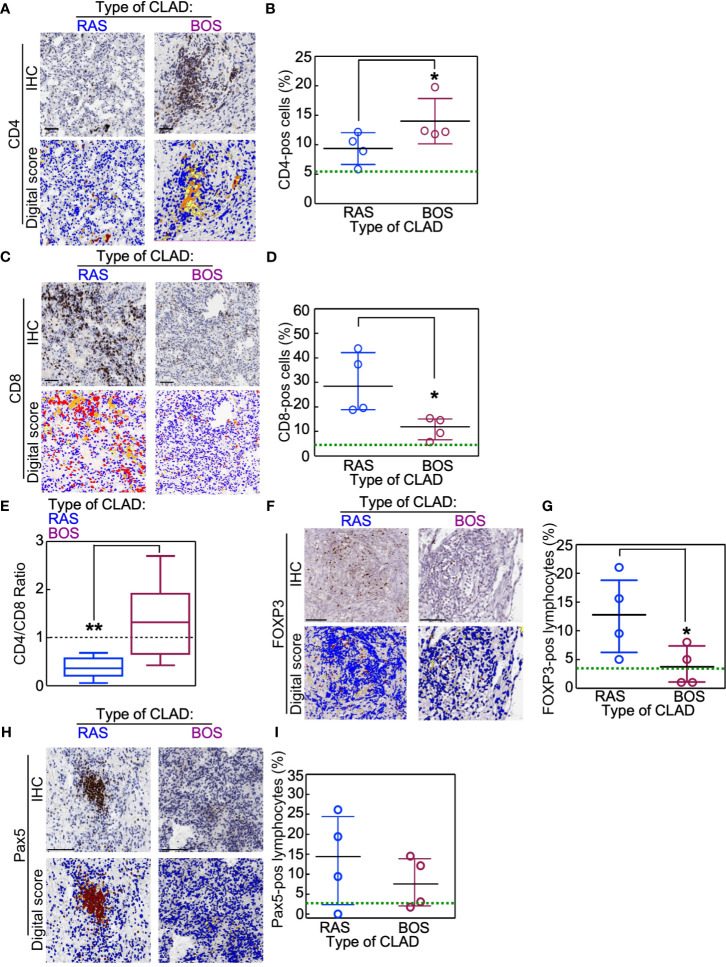
Immunophenotype of lungs explanted for re-transplantation (re-LTx). Lymphocyte subsets were analyzed in BOS and RAS re-LTx (n = 4 cases per condition). **(A–D)** CD4^pos^ **(A, B)** and CD8^pos^ **(C, D)** T lymphocytes were identified and scored as the percentage of positive cells in that area using Aperio algorithm (digital score mask). Each dot is a case, and lines indicate median with IQR. *p = 0.02 by Mann-Whitney U test. **(E)** The CD4/CD8 ratio was calculated for RAS and BOS re-LTx. Data are expressed as box-plot with whiskers indicating min to max values. **p = 0.003 by two-sided Mann-Whitney U test. **(F–I)** Foxp3-positive **(F, G)** or Pax5-positive (B cells; **H, I**) lymphocytes were identified and scored in RAS or BOS lungs. Each dot is a case, and lines indicate median with IQR. *p = 0.03 by Mann-Whitney U test. Scale bars, 100 μm. Green lines within graphs indicate the mean value of the marker measured in normal lungs (see also **Supplementary Figure 3** for details).

Data obtained upon analyzing lymphocyte subpopulations were compared using non-parametric Mann-Whitney U tests. Results showed that, as compared to BOS grafts, RAS grafts were characterized by a reduced CD4 ^pos^ T lymphocytic infiltrate (BOS *vs*. Ras: p = 0.02; **Figures 1A, B**) and a predominant presence of CD8^pos^ T lymphocytes (BOS *vs*. Ras: p = 0.02; **Figures 1C, D**) that resulted in the inversion of the CD4/CD8 ratio (BOS *vs*. Ras: p = 0.003; **Figure 1E**). These quantifications of the lymphocytic infiltrates were similar in the stromal or alveolar compartments of BOS and RAS lungs (**Supplementary Figure 1**). These differences were not related to a different number of lymphoid follicles present in the lung parenchyma of BOS and RAS lungs, since neither the number nor the area of the lymphoid follicles was different in the two CLAD types (**Supplementary Figure 2**).

RAS grafts were also characterized by a higher percentage of Foxp3^pos^ lymphocytes (BOS *vs.* Ras: p = 0.03; **Figures 1F, G**) and by increased amounts of B-cells (Pax5^pos^-cells; **Figures 1H, I**), even if this difference approached but did not reach statistical significance. On the contrary, CD57-expresssing NK cells were not detected in the lymphocytic infiltrates of either BOS or RAS lungs (data not shown). Analysis of these markers in normal lungs showed that, in physiologic conditions, these factors are poorly present within the lung parenchyma (**Supplementary Figure 3**).

**Figure 2:**
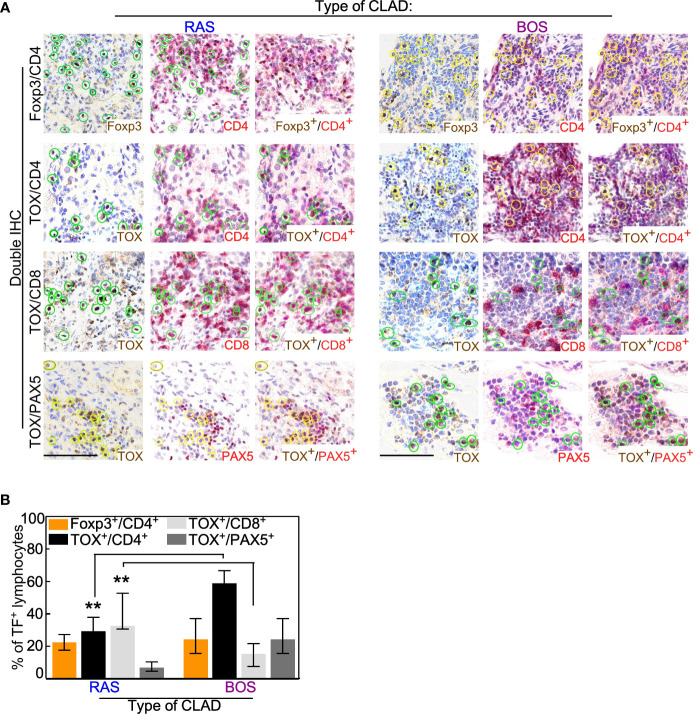
Co-expression analysis of lymphocytic lineage markers. **(A)** Double IHC was performed with the lineage-specific transcription factor (TF) Foxp3 or TOX and the membrane antigens CD4, CD8, or Pax5. Representative images of the indicated staining are shown for RAS and BOS lung explants. Scale bars, 100 μm. See also **Supplementary Figure 1**. **(B)** The percentage of lymphocytes positive for the indicated membrane antigen was calculated from the total number of cells expressing Foxp3 or TOX. Bars represent median ± IQR. **p = 0.008 by two-sided Mann-Whitney U test.

Co-expression analyses of cell lineages markers showed that RAS grafts were enriched in exhausted CD8^pos^ T cells (BOS *vs*. Ras: p = 0.008), whereas a higher amount of exhausted CD4^pos^ T cells and B cells (Pax5^pos^/TOX ^pos^) was seen in BOS grafts (BOS *vs*. Ras: p = 0.008; **Figures 2A, B** and **Supplementary Figure 1**). In contrast with these results, the percentage of Tregs (CD4 ^pos^/Foxp3 ^pos^) lymphocytes was comparable in the two CLAD phenotypes (**Figures 2A, B** and **Supplementary Figure 4**)

**Figure 3:**
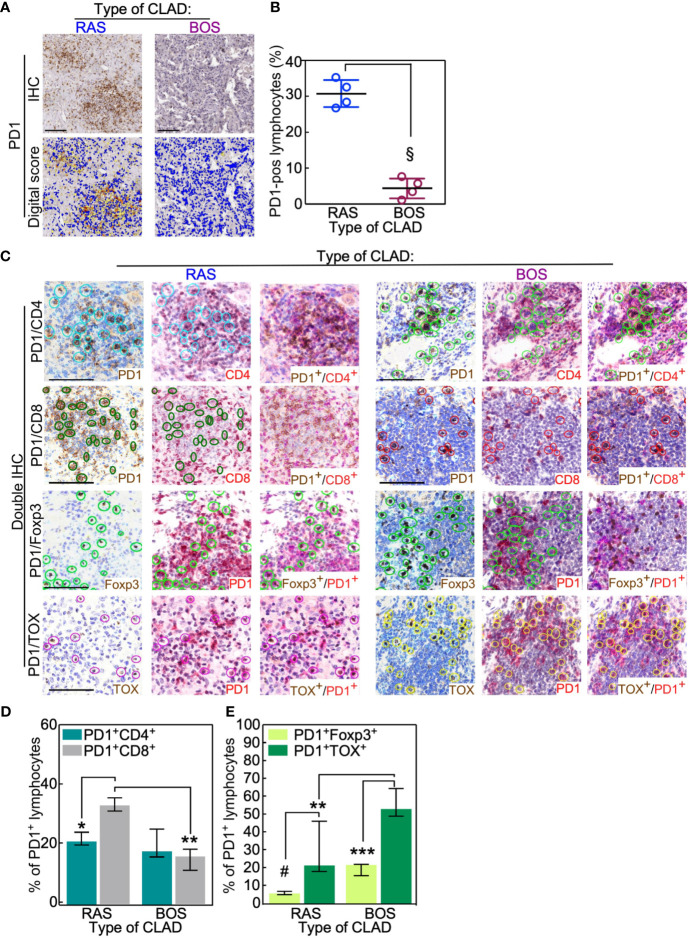
RAS lungs have fewer exhausted PD1-positive T cells than BOS lungs. **(A, B)** PD1-positive lymphocytes were analyzed in BOS and RAS re-LTx and scored as the percentage of positive cells in that area using Aperio algorithm (digital score mask). Each dot is a case, and lines indicate median with IQR. §, p = 0.02 by two-sided Mann-Whitney U test. **(C–E)** Double IHC staining was performed in RAS and BOS re-LTx with PD1 and either CD4 or CD8, the transcription factor Foxp3, or TOX. The percentage of lymphocytes positive for the membrane antigens (CD4, CD8; D) or the nuclear antigens **(E)** was calculated from PD1-positive cells. See also **Supplementary Figure 3**. Bars represent median ± IQR. *p = 0.01; **p = 0.0007 in **(D)** and **p = 0.003; ***p = 0.0005 in **(E)**; ^#^p = 0.006 by two-sided Mann-Whitney U test. Scale bars, 100 μm.

Notably, the lymphocytic infiltrate of RAS grafts was characterized by higher presence of PD1-positive cells (**Figures 3A, B**) compared to BOS grafts (BOS *vs*. Ras: p = 0.02). A more frequent expression of the other immune checkpoint molecules PDL1 and CTLA4 (**Supplementary Figures 5A–D**) was seen in the same RAS grafts. In control lung parenchyma, no PD1 expression could be detected except for histiocytes (**Supplementary Figure 6**).

Double IHC staining with PD-1 and CD4 or CD8 showed that PD-1^pos^/CD8^pos^ T lymphocytes were more abundant in RAS than in BOS lungs (BOS *vs*. Ras: p = 0.01; **Figures 3C, D** and **Supplementary Figure 7**). Further, co-expression analysis of PD1 with the transcription factors Foxp3 or TOX indicated that PD-1^pos^ lymphocytes (i.e., exhausted T cells) were reduced in RAS compared to BOS grafts (BOS *vs*. Ras: p = 0.003; **Figures 3C, E** and **Supplementary Figure 7**). This observation was further supported by analysis of TIGIT; indeed, TIGIT expression was predominantly detected in RAS grafts infiltrates, and TIGIT-positive cells were in the same area of CD8- and TOX-expressing lymphocytes (**Supplementary Figures 5E, F**). These data indicate that RAS grafts are characterized by a significantly reduced population of exhausted (PD-1^pos^/TOX^pos^), in particular, of exhausted Treg (PD-1^pos^/FOXP3 ^pos^) T lymphocytes.

Taken together, these data suggest that the RAS form of CLAD is characterized by a higher cytotoxic T-cell response (PD-1^pos^/CD8^pos^) and by a reduced presence of Treg lymphocytes. Conversely, a predominant CD4^pos^ T-cell infiltrate and increased amounts of exhausted and exhausted Treg cells are seen in BOS lungs.

## Discussion

Since 1992, researchers have analyzed the effects of programmed death 1 receptor and its ligands in balancing T-cell activation and tolerance (14). The current observational study reports the first results obtained by analyzing immune checkpoints expression in chronic lung rejection.

Main result herein is that the immune cell infiltrates of BOS and RAS are different. Thus, PD-1-, PD-L1-, and CTLA4-expressing T lymphocytes were significantly increased in explanted lungs of patients who developed the RAS type of CLAD. Notably, PD-1^pos^/TOX^pos^ (exhausted) and PD-1^pos^/FOXP3^pos^ (exhausted regulatory) T lymphocytes were significantly reduced as well in RAS compared to BOS grafts. Taken together, these results allow the speculation that tolerance-inducing mechanisms are particularly defective in RAS, offering a possible immunological explanation that justifies the severity of this CLAD phenotype.

Moreover, we found a pathological inversion of the CD4/CD8 ratio in the lymphocytic infiltrate of RAS grafts. The inversion of the CD4/CD8 T lymphocyte ratio was shown to be associated with higher rejection grade and shorter survival in the setting of kidney and cardiac allograft, but has never been described in LTx (15, 16).

Recent results obtained by reviewing the outcome of 608 LTx recipients who were transplanted between 2001 and 2015 showed that, out of 268 patients who developed CLAD, 47 had RAS, whereas 215 had BOS. Median survival for RAS and BOS cohort was 1.2 and 7.2 years, respectively (17). Our data indicate that the immune scenario that characterizes these two CLAD phenotypes is clearly different and could offer an explanation for the diverse outcomes associated with RAS and BOS CLAD. Thus, we suggest that the peculiar reduction of exhausted T lymphocytes, together with the increased presence of checkpoint-expressing cells, justifies the worst prognosis that characterizes the RAS phenotype of CLAD.

T-cell exhaustion is the result of chronic, prolonged antigenic stimulation and is characterized by the loss of cytokine production and by apoptotic T-cell death. This phenomenon plays a deleterious role in chronic infections and cancer, where disease progression is associated with the waning of immune responses (18, 19). On the other hand, T-cell exhaustion results in self-tolerance and such T-cell condition is associated with transplant tolerance (20–22). The observation that exhausted Treg (PD-1^pos^/Foxp3^pos^) lymphocytes were greatly reduced in RAS lungs could thus provide a preliminary immunological explanation for the clinical observation that RAS is the worst CLAD phenotype. On the other hand, PD-1 is a membrane protein involved in the induction of cell death and in the inhibition of cell proliferation and cell cycle progression. By controlling the magnitude of T-cell responses, PD-1 protects against self-reactivity, leading to induction of tolerance (23–26). PD-1 binds to PDL1, and the PD1/PDL1 pathway was shown to be involved in the regulation of immune responses in pathological and physiological scenarios (27–32); CTLA-4 has a similar dampening effect on immune responses upon binding CD80 and CD86. We observed that PD-1 as well as PD-L1- and CTLA-4-expressing T lymphocytes were increased in RAS lung. This could be explained as a futile attempt, possibly rendered even more helpless by the simultaneous reduction of Treg lymphocytes and the increase of cytotoxic T-lymphocytes, to prevent organ rejection *via* the inhibition of cell-mediated immune responses in RAS CLAD. Notably, our preliminary results offer support to recent data showing that the presence of PD-L1-expressing T lymphocytes in heart transplanted biopsies is predictive of organ rejection (33), and raise the possibility that modulation of PD-1 activity could be useful in preventing graft rejection. It is worth noting that an indirect confirmation of this possibility comes from heart-transplanted recipients who developed malignancies: in these patients, immune checkpoint inhibitor-based therapies resulted in rejection (8).

Generalization of our results is limited by the single center nature of our study and by the limited sample size: this limitation is nevertheless difficult to overcome because re-transplantation is a scarcely practiced procedure (less than 5% of total LTx worldwide). These considerations notwithstanding, we underline that all the consecutive patients that received re-LTx for CLAD in a 6-year period were analyzed to avoid any selection bias. More analyses on ampler cohorts of patients, as well as analyses performed on lung biopsies and fresh specimens, will be needed to lend support to our preliminary results. As far as the diagnosis of rejection is concerned, it would be extremely useful to analyze whether the immune proteins related to CLAD could be early markers of rejection in lung graft biopsies. Further, in the future, the potential of analyzing PD1-positive lymphocytes in the BAL fluids may represent an additional tool for the clinical follow-up of LTx patients.

Cancer therapy has been revolutionized by the use of checkpoint antagonists; the clarification of the role of these molecules in organ transplantation could lead to the design of novel therapeutic options to improve the prognosis of solid organ transplantation. Result herein could offer initial support to the hypothesis that the modulation of immune checkpoint molecules might be useful to reach such objective.

## Data Availability Statement

The original contributions presented in the study are included in the article/**Supplementary Material**. Further inquiries can be directed to the corresponding authors.

## Ethics Statement

The studies involving human participants were reviewed and approved by Review Board of Fondazione IRCCS Ca’ Granda Ospedale Maggiore Policlinico (ref. 1693/2018). The patients/participants provided their written informed consent to participate in this study.

## Author Contributions

IR conceived the study, performed surgical procedures and co-wrote the manuscript. VV designed the experiments, performed the immunohistochemical analyses, and co-wrote the manuscript. IR and VV share first authorship. LM and VR were responsible for the clinical follow up of patients. GC performed the immunohistochemical analyses. FB and SF designed the study and co-wrote the paper. MN performed surgical procedures and co-wrote the manuscript. LR performed surgical procedures and co-wrote the manuscript. MC conceived the study and the experiments and wrote the manuscript. All authors contributed to the article and approved the submitted version.

## Conflict of Interest

The authors declare that the research was conducted in the absence of any commercial or financial relationships that could be construed as a potential conflict of interest.

## Publisher’s Note

All claims expressed in this article are solely those of the authors and do not necessarily represent those of their affiliated organizations, or those of the publisher, the editors and the reviewers. Any product that may be evaluated in this article, or claim that may be made by its manufacturer, is not guaranteed or endorsed by the publisher.

## Footnotes

1 BOS Bronchiolitis Obliterans Syndrome; CF Cystic Fibrosis; CLAD Chronic Lung Allograft Disease; LCH Pulmonary Langerhans Cell Granulomatosis - Histiocytosis X; RAS Restrictive Allograft Syndrome
