# Supplementary material for: Immune Checkpoints Expression in Chronic Lung Allograft Rejection
Source: Front Immunol. 2021 Aug 13;12:714132. doi: 10.3389/fimmu.2021.714132 (PMC8418069; doi:10.3389/fimmu.2021.714132)
Supplement: Supplementary file 1 [file DataSheet_1.docx]

**Immune Checkpoints Expression in Chronic Lung Allograft Rejection**

Ilaria Righi, Valentina Vaira, Letizia Corinna Morlacchi, Giorgio Alberto Croci, Valeria Rossetti, Francesco Blasi, Stefano Ferrero, Mario Nosotti, Lorenzo Rosso, Mario Clerici.

**Supplementary Information**

**Supplementary Table 1**

| Primary antibodies used for immunohistochemistry |
| --- |
| - PD1/CD279 (clone NAT105; Ventana Medical Systems, part of Roche Diagnostics, Monza, Italy) - PDL1/CD274 (clone 22C3; Dako, Agilent Technologies Inc., Santa Clara, CA, USA) - CTLA4/CD152 (TA322656; OriGene Technologies, Rockville, MD, USA) - CD4 (clone 4B12; Dako) - CD8 (clone C8/144B; Dako) - hFoxp3 (MAB8214; R&D Systems Inc., Minneapolis, MN, USA) - TIGIT (LS-C669093; LSBio, Seattle, WA, USA) - CD57 (clone TB01; Dako) - TOX (HPA018322; Sigma Aldrich, Merck KGaA, Darmstadt, Germany) - B-Cell-Specific Activator Protein (Pax5, Clone DAK-Pax5; Dako) |

**Supplementary Table 2.** The mean number of cells considered to count each of the indicated markers is shown for both BOS and RAS cases.

|  | BOS | | | | RAS | | | |
| --- | --- | --- | --- | --- | --- | --- | --- | --- |
|  | **CD4** | **CD8** | **FOXP3** | **PAX5** | **CD4** | **CD8** | **FOXP3** | **PAX5** |
| Mean | 2050 | 2471 | 1406 | 1104 | 2435 | 2047 | 1670 | 1287 |
| Lower 95% CI of mean | 1199 | 2231 | 438.1 | 696.5 | 1504 | 1734 | 751.1 | 777 |
| Upper 95% CI of mean | 2901 | 2711 | 2374 | 1511 | 3365 | 2360 | 2589 | 1797 |

**Supplementary Figures**

**
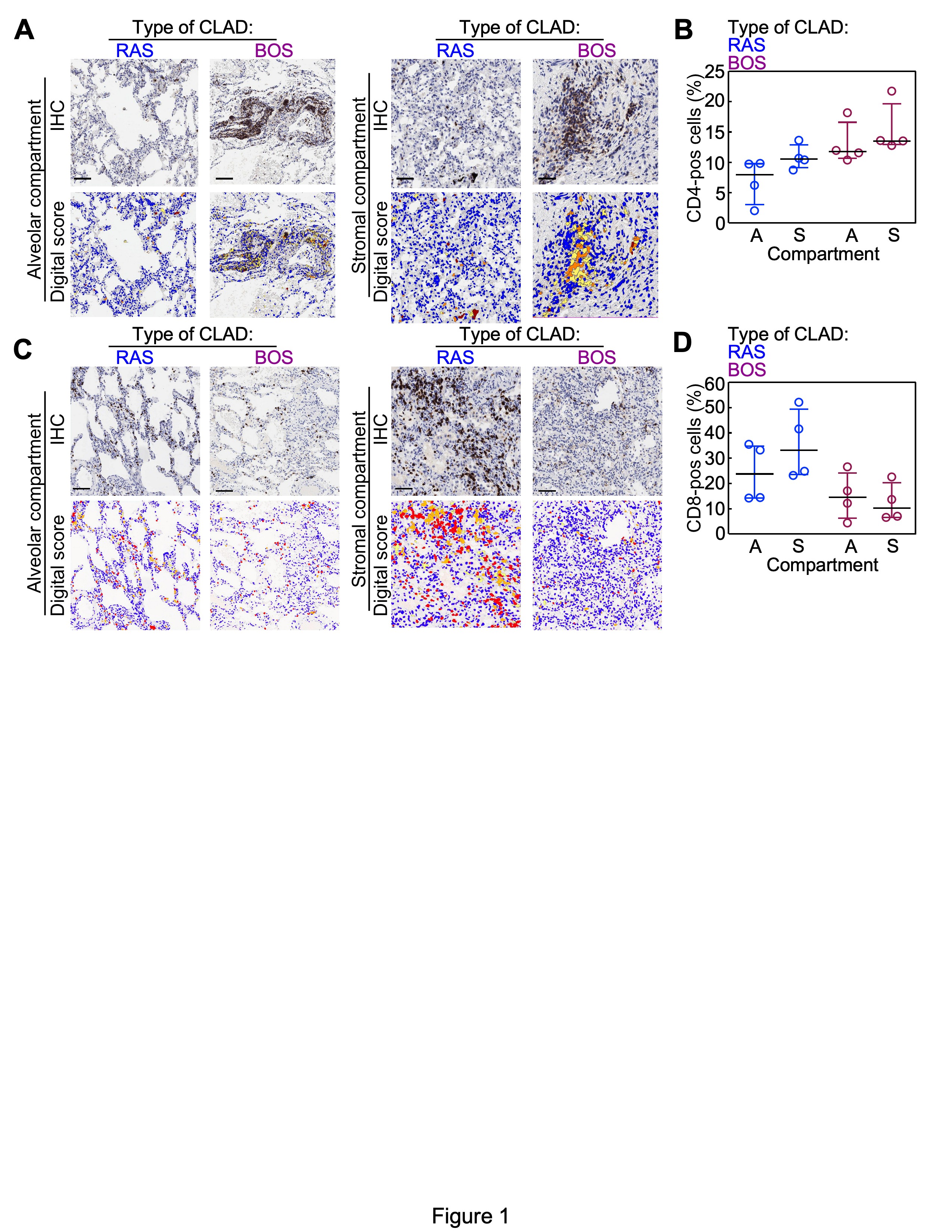
**

**Supplementary Figure 1.** Details of the immunophenotype of lungs explanted for re-transplantation (re-LTx). CD4^pos^ (A,B), and CD8^pos^ (C,D) T-Lymphocytes analyzed separately in the alveolar (A) or stromal (S) compartment of BOS and RAS re-LTx and scored as the percentage of positive cells in that area using Aperio algorithm (digital score mask). Each dot is a case and lines indicate median with IQR.

**
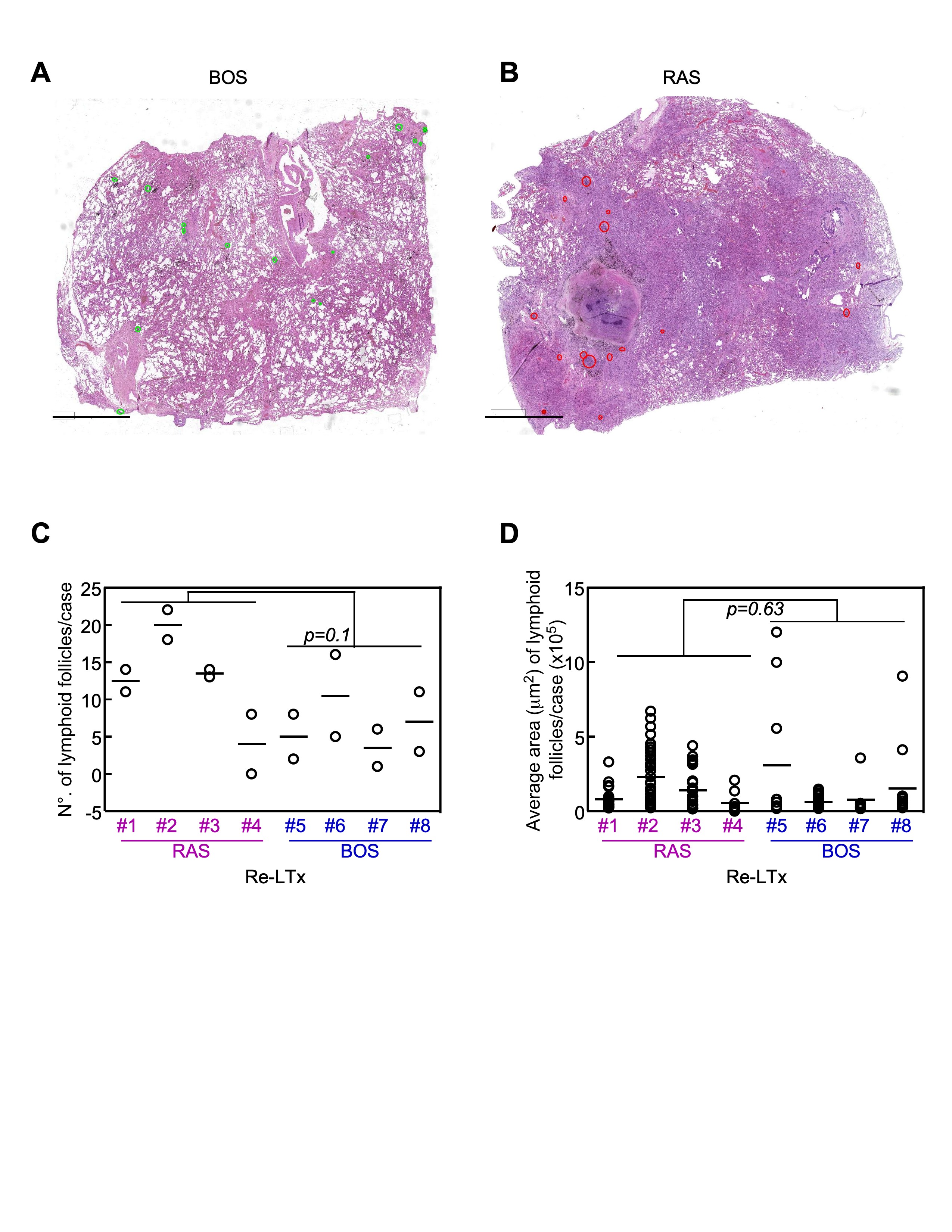
**

**Supplementary Figure 2.** Analysis of lymphoid follicles in BOS and RAS lungs. Two lung blocks per case were analyzed for lymphoid follicles presence. **A,B)** Representative full sections of BOS (**A**) and RAS (**B**) lung tissues with indicated lymphoid follicles (circles). Scale bars, 5mm.**C,D**), Quantification of the number (**C**) and area (**D**) of lymphoid follicles detected in BOS and RAS lungs. Each dot is a measure and line represents the mean level.

**
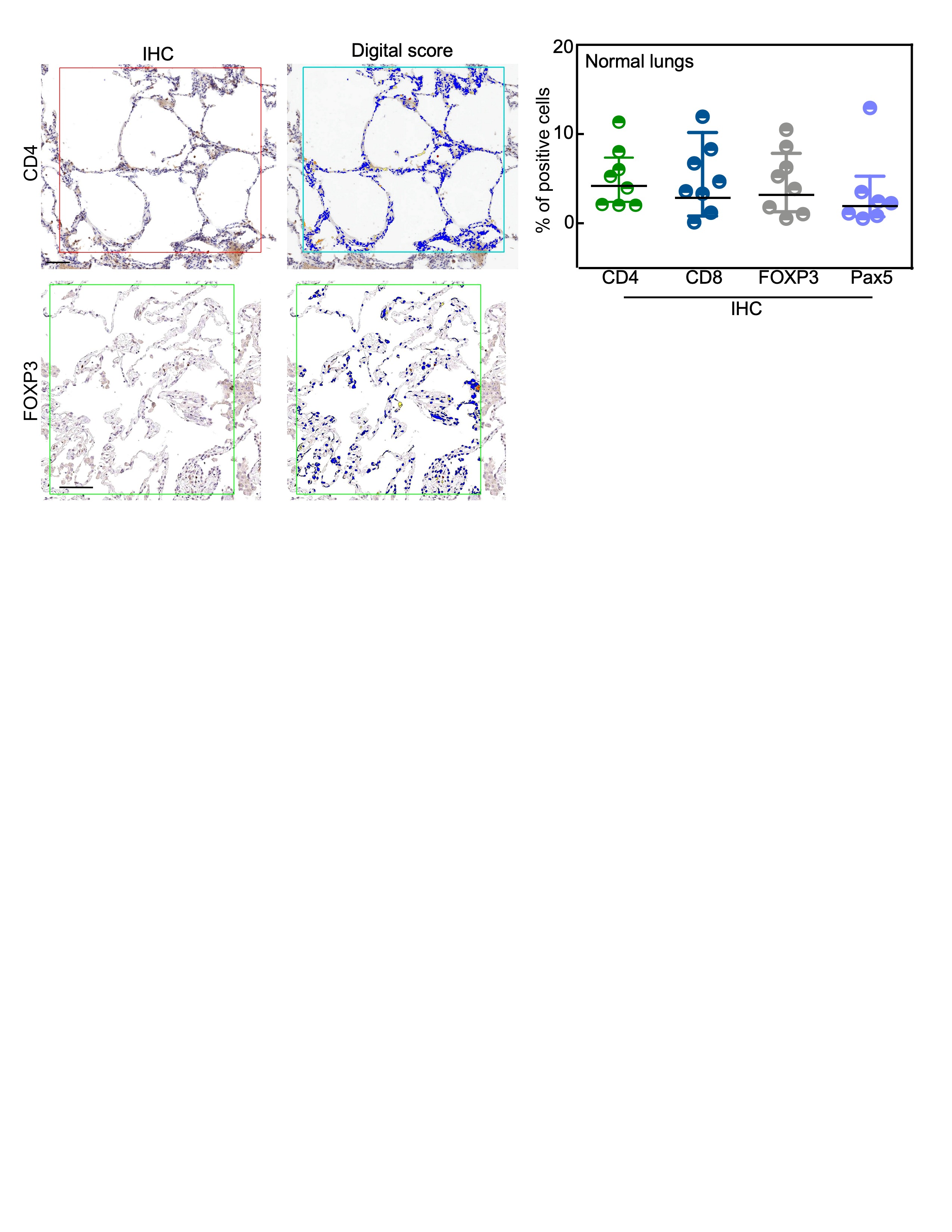
**

**Supplementary Figure 3.** Analysis of the indicated immune-related molecules in normal lungs. Representative images are shown for CD4 and FOXP3 presence (left panels); *right panel*, quantification of the markers using the ImageScope algorithm. Scale bars, 100μm.

**
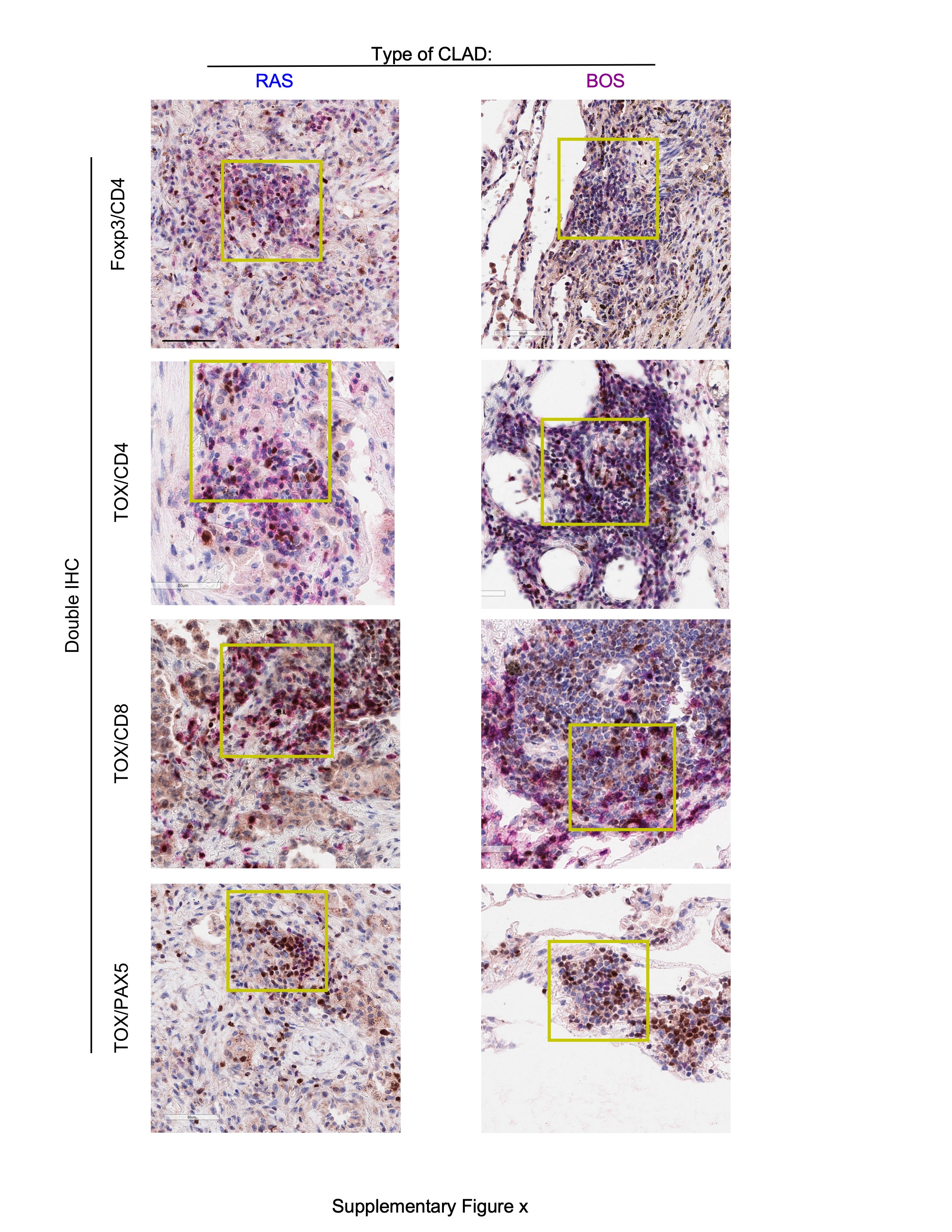
**

**Supplementary Figure 4.** Double IHC of the lineages markers CD4, CD8 and Pax5 with the transcription factors FOXP3 or TOX. Unprocessed images relative to Figure 2 are shown. Yellow insets indicate the part of the image shown in Figure 2. Scale bars, 100 μm.


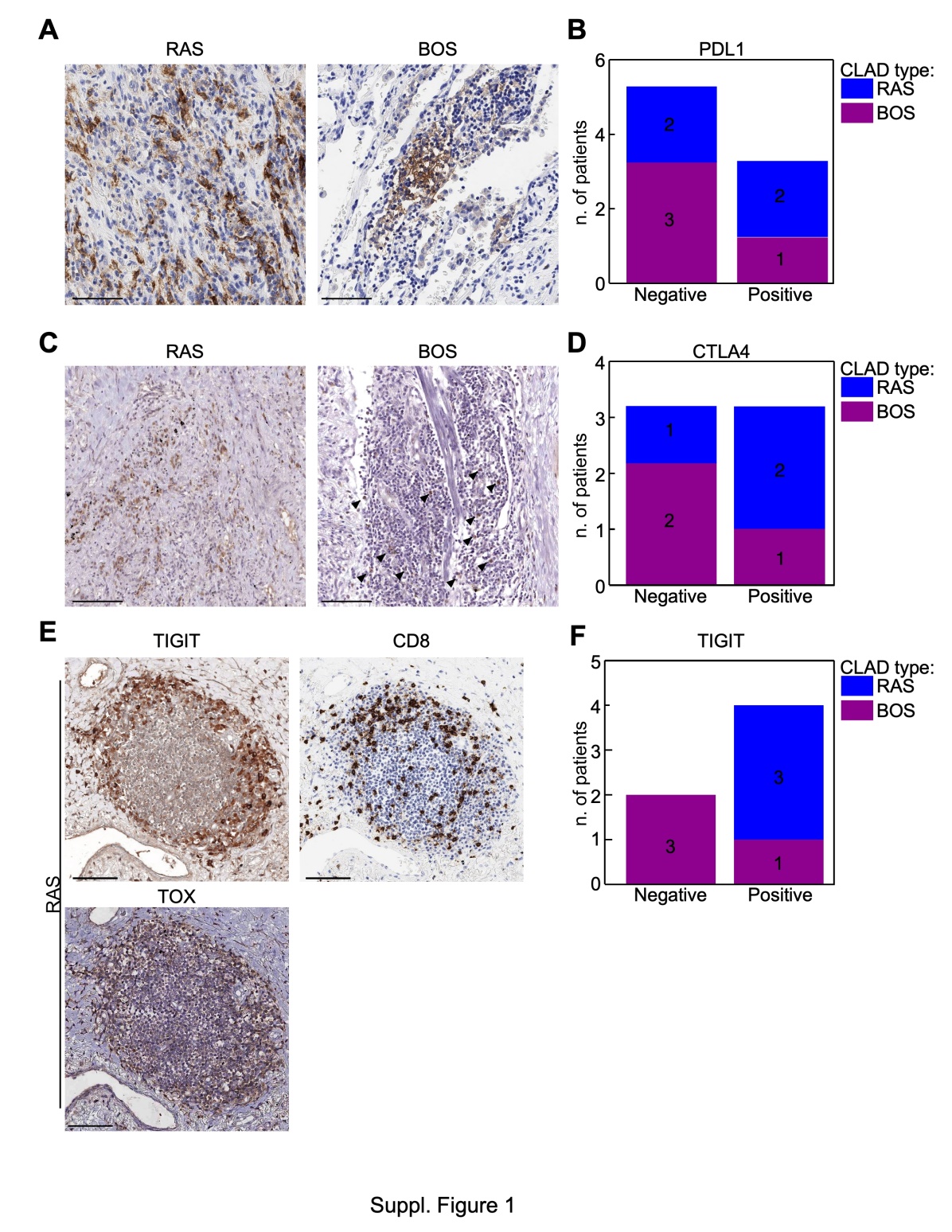


**Supplementary Figure 5.** Immune checkpoints expression in re-LTx lungs. Presence (>5% of positive lymphocytes) of PDL1 (**A,B**), or CTLA4 (**C,D**) was analyzed in RAS and BOS lungs by immunohistochemistry and the number of patients positive for the indicted marker is shown (**B,D**). For CTLA4, IHC was not evaluable in two patients. **E**) TIGIT expression was evaluated in RAS and BOS lungs in the same area where CD8- and TOX-positive lymphocytes were detected. Representative images of a RAS case are shown. **F)** TIGIT presence was scored in re-LTx lungs. In one case IHC was not evaluable. Scale bars, 100 μm.


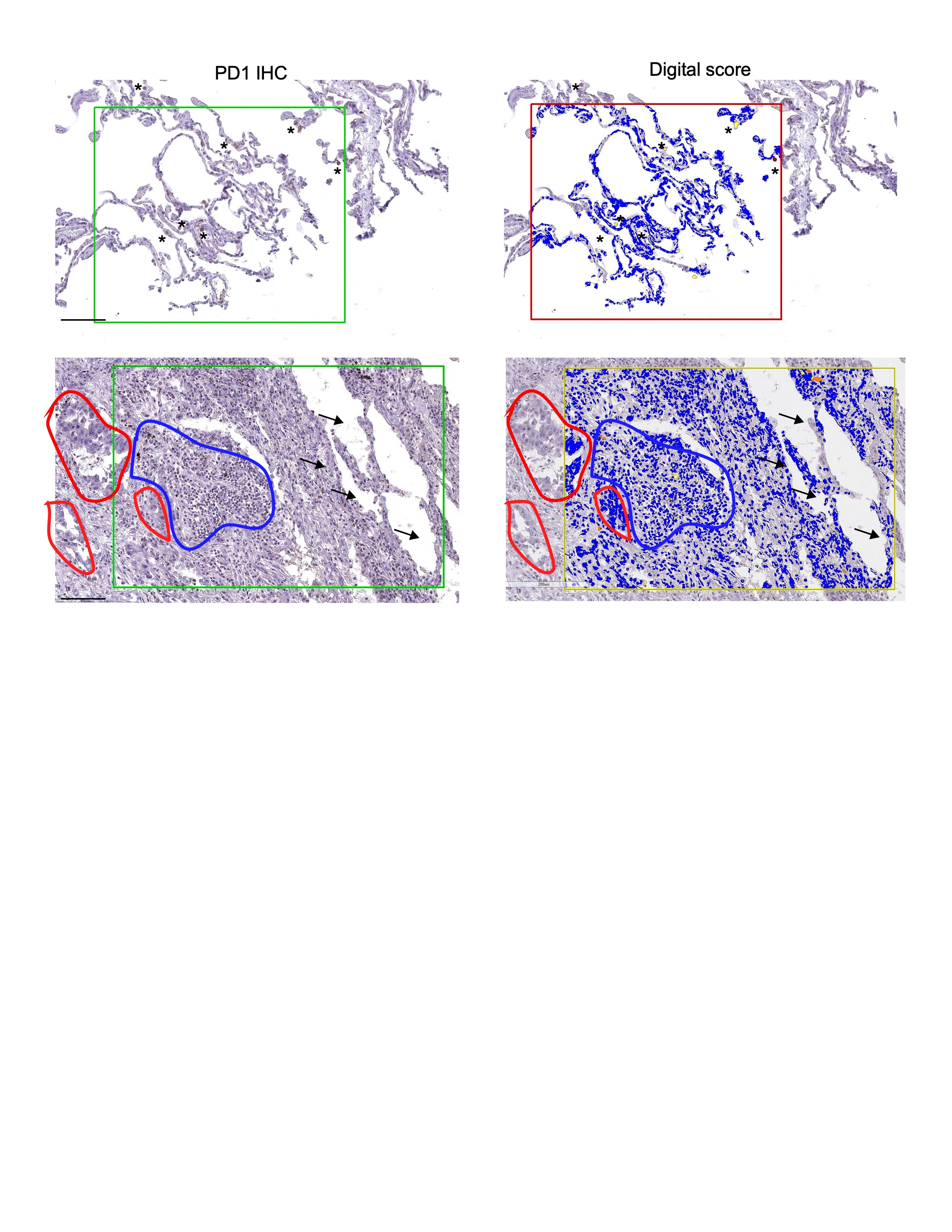


**Supplementary Figure 6.** PD1-positive lymphocytes are absent in normal lung parenchyma. PD1 immunohistochemistry was performed in normal lungs; upper panels, representative images of normal parenchyma with PD1-positive histiocytes (stars). Lower panels, as positive control we stained a lung cancer with normal parenchyma in the same section with the PD1-specific antibody. Red-circled area, tumor glands; blue-circled-areas: PD1-positive tumor-associated lymphocytes; arrows: normal lung parenchyma. Scale bars, 100 μm.

**
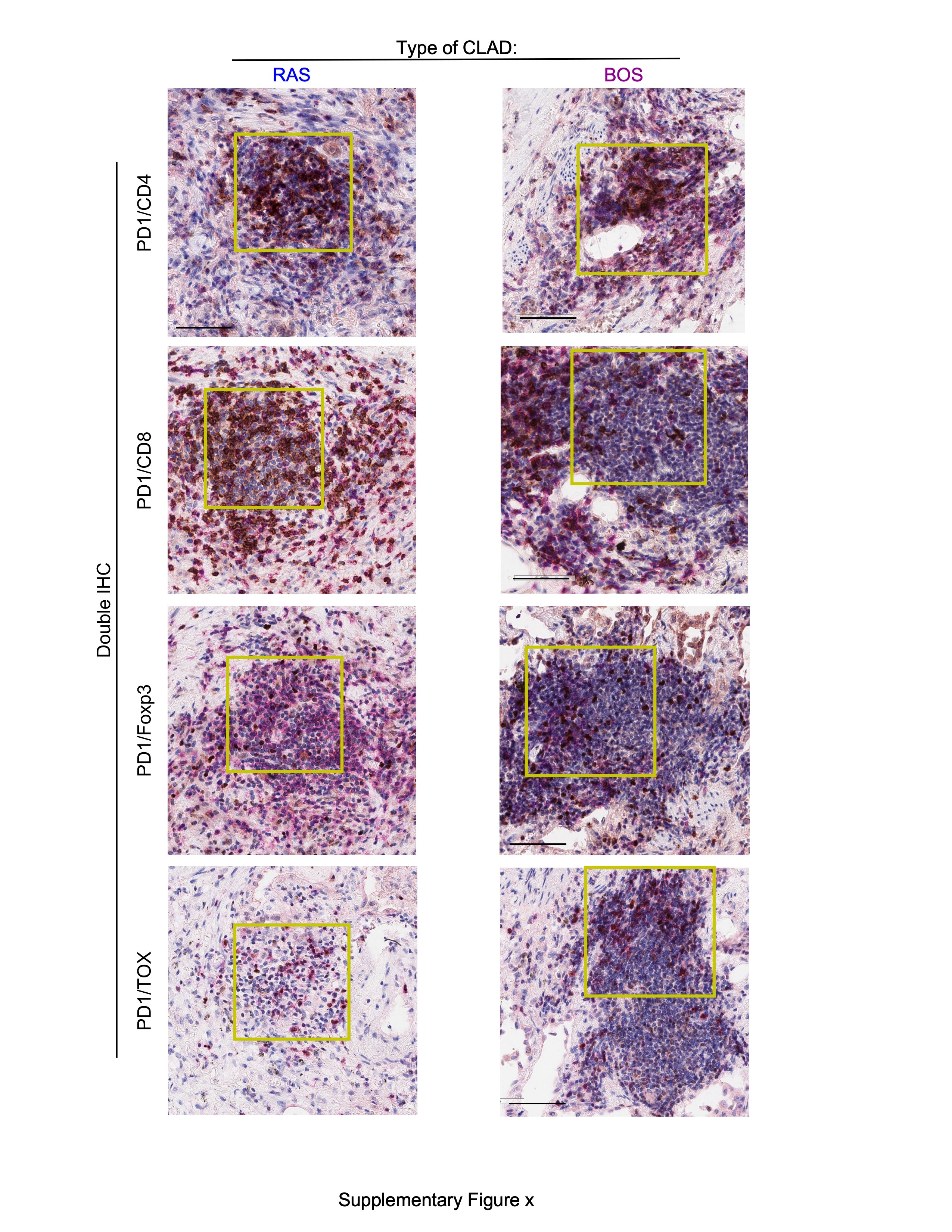
**

**Supplementary Figure 7.** Characterization of PD1-positive lymphocytes in RAS or BOS lungs: double IHC for PD1 with either the lineages markers CD4, CD8 or with the transcription factors FOXP3 or TOX was preformed. Unprocessed images relative to Figure 3 are shown. Yellow insets indicate the part of the image shown in Figure 2. Scale bars, 100 μm.
